# The role of anatomy education in strengthening healthcare in Rwanda

**DOI:** 10.1097/MS9.0000000000003287

**Published:** 2025-04-10

**Authors:** Benimana Darius, Shema Auverdin, Igiraneza Divin Jonathan, Ndayishimiye Pacifique, Ishimwe Ange Chifra, Niyibikora Fabrice, Tuyishime Moise, Nezerwa Dieudonne, Izere Salomon

**Affiliations:** Department of General Medicine and Surgery College of Medicine and Health Sciences, University of Rwanda, Kigali, Rwanda

**Keywords:** anatomy education, healthcare system, medical training, Rwanda

## Abstract

**Background:**

Anatomy education is a cornerstone in medical training, providing foundational knowledge essential for effective diagnosis, treatment, and surgical precision. Since its introduction in Rwandan medical education in 1970, significant strides have been made, including the adoption of cadaver-based learning and modern technological tools. Despite these advancements, challenges such as limited resources, high student-to-staff ratios, and insufficient engagement in anatomy learning persist, hindering optimal healthcare delivery.

**Objective:**

This review evaluates the role of improved anatomy education in strengthening the Rwandan healthcare system and highlights strategies for enhancing its impact.

**Methodology:**

A narrative review approach was adopted, utilizing secondary data from peer-reviewed journals accessed through PubMed, Google Scholar, Embase, Scopus, WHO databases, and other sources. Articles published in English within the last 5 years were included to ensure relevance and accuracy.

**Results:**

Anatomy education in Rwanda has evolved significantly, with the integration of cadaver dissection, radiological anatomy, and electronic tools into medical training curricula. However, the lack of sufficient cadavers, modern teaching facilities, and resources continues to impede progress. Evidence suggests that innovative teaching strategies and mentorship can foster greater interest in surgical fields among medical students, addressing critical gaps in the healthcare workforce.

**Conclusion:**

Strengthening anatomy education is pivotal to enhancing individual competencies and addressing workforce shortages in Rwanda’s healthcare system. Continuous investment in resources, innovative teaching methods, and active student engagement are essential for building a skilled healthcare workforce capable of meeting the nation’s health demands.

## Introduction

Anatomy, in its entirety, makes up a vital portion of the surgical field and the health sector at large. A deep understanding of anatomy is essential for safe clinical practice and identifying critical landmarks for surgery. Rwandan medical schools have recently implemented a holistic approach to learning in anatomical modules. Cadaver-based approaches, random assessment tests, radiology anatomy, and electronic anatomage tools make up anatomy education in Rwandan medical schools^[1]^. From the first training of anatomists and anatomy laboratory technicians at the University of Rwanda (UR) in 2002, medical universities have seen progressive growth in the technical exposure to the necessity of anatomy and shaping future medical doctors and anatomists in different departments in the health sector^[2]^.

Anatomy education encompasses the use of different anatomical landmarks and tools to shape future healthcare providers. In Rwanda, three medical schools provide anatomy education in their respective medical classes. This number of medical schools available in Rwanda contributes to the government’s 4-in-4 program of quadrupling medical graduates in the upcoming 4 years. Nonetheless, recent data published by the Ministry of Health in 2019 illustrates that there were 21 679 health professionals in Rwanda by 2018, with only 1648 (8%) being medical doctors and 62 surgeons among them. These numbers demonstrate the necessity of profound mentoring of medical students willing to pursue a career in the surgical field^[3]^.

**HIGHLIGHTS**

- Anatomy is essential in healthcare, supporting accurate diagnoses, safe surgeries, and effective patient management.
- Anatomy education in Rwanda has evolved since the early 1970s, starting with Belgian faculty. It progressed to cadaver-based learning and now incorporates advanced resources like digital anatomy tools and radiological imaging techniques.
- Anatomy education faces challenges affecting effective learning. Limited cadaver access and a shortage of advanced teaching tools complicate practical education. Rapid enrollment growth has led to overcrowded classrooms and labs, impacting training quality.
- Integrating team-based learning and peer-learning programs is encouraged to foster collaborative environments. Cutting-edge tools like three dimensional (3D) anatomical atlases and virtual dissection platforms are being introduced to enhance understanding of anatomical concepts.
- It is essential to invest in advanced anatomy labs and digital resources, integrate clinicians into teaching for practical, application-focused learning, and establish mentorship programs to inspire and guide students toward careers in anatomy-related fields.

A recent study conducted by Carmichael *et al*^[4]^ showed that medical students can boost their passion for anatomy and its related fields through cadaver-based sessions. In this study, anatomists and surgical residents co-facilitated a 1-hour session that included common surgical cases and prompted critical thinking and problem-solving skills. After the session, 83 students completed a post-course survey, and the majority highlighted the importance of anatomy education. They also agreed that the session had boosted their interest in surgery. This study was necessary to illustrate the necessity of medical students’ exposure to anatomy education and the fact that mentorship will nurture the passion and interest in medicine regarding anatomy and its related fields, such as surgery^[4]^.

According to Asante *et al*^[5]^, it was suggested that there is not only a one-way approach to the best teaching method, but the association of different methods would be complementary. For example, full-body dissection would be the best approach for medical students, especially those interested in surgical careers^[5]^. Even though dissection is essential as a teaching approach, it must be complemented with other learning methods necessary to strengthen Rwanda’s healthcare^[1]^. This review, therefore, intends to illustrate the impact that medical education has and can have on the healthcare sector in Rwanda.

## Background and context of anatomy learning in Rwanda

Anatomy was introduced into the medical program in Rwanda 6 years after the beginning of medical education. This happened during the academic year 1970–1971. Initially, anatomy, along with other fundamental biomedical sciences, was taught by professors from Belgium^[2]^. The clinical anatomy unit started its development in 2002. Initially, anatomical specimens were collected from amputated limbs and body organs from autopsies. A law that regulates the use of human bodies, organs, and tissues in teaching and research was promulgated in 2010, and a MoU on the supply of cadavers for teaching purposes with various hospitals in Rwanda. In 1983, Rwandan surgeons began taking over anatomy teaching, significantly shifting the educational landscape^[2]^.

The UR’s CMHS offers a 6-year medical program. Within this program, there are three modules related to human anatomy. First is Human Anatomy I, which covers the limbs, back, and spinal cord. The second is Human Anatomy II, which is based on the thorax, abdomen, pelvis, and perineum, and Human Anatomy III, which focuses on the head, neck, and neuroanatomy^[2]^.

Anatomy in Rwanda has come a long way, starting with the collection of anatomical specimens from amputated limbs and autopsies. The development of the clinical anatomy unit in Rwanda began in 2002, and efforts have been guided by a commitment to deliver anatomy relevant to clinical practice. The use of cadaver dissection remains a gold standard, and Rwanda has established strategies for acquiring, processing, and using cadavers for teaching purposes. In 2020, the Society of Clinical Anatomy of Rwanda was founded to promote anatomical sciences and create a community of anatomy professionals in the country^[2]^.

## Importance of anatomy education in healthcare delivery

Anatomy studies the physical structure of humans, animals, and other living organisms, particularly as evident through dissection and the analysis of separate parts. Anatomy education aims to equip medical and health-related students with knowledge of human body structures and their locations. It is the fundamental cornerstone healthcare professionals must have in diagnosing, treating, and caring for patients effectively daily^[6]^.

Anatomy is paramount to medical graduates in making accurate treatments, diagnoses, medical imaging interpretation, and surgical procedures. A strong understanding will enable a physician to conduct a thorough physical examination, identifying abnormalities and determining the root cause of symptoms. It is also interrelated with physiology and pathology; it makes it easy for medical professionals to determine the scope of their specialization, such as orthopedics, general surgery, and cardiology^[7]^.

Surgical anatomy distinguishes anatomical position and direction and reveals anatomical variations, connections to nearby structures, and disease-induced alterations in normal anatomy. As surgical treatments advance, a better understanding of anatomy is required to pursue new areas of research and therapy because it acts as a roadmap for most surgeons. New imaging modalities, endoscopic surgical procedures, and developments in minimally invasive surgery require a different understanding of anatomy than traditional surgical methods. Its use makes surgical treatments safer and more successful by guiding doctors to avoid injuring critical tissues such as nerves^[8]^.

Anatomy knowledge is required for healthcare practitioners to comprehend how the body is structured and works. This plays a vital role in making clinical decisions using endoscopy, laparoscopy, computer tomography, magnetic resonance imaging, and other 3D visualization technologies^[9]^.

The rise of these advanced procedures corresponded with the development of minimally invasive therapies targeting specific organs and places within the body.

Providing an accurate diagnosis and appropriate patient care without understanding anatomy is extremely difficult. A gastroenterologist, for example, must have a detailed awareness of the nature of the small intestines and the organs surrounding them to construct a suitable rehabilitation program for a patient recovering from an intestinal hernia (obstruction)^[10]^.

## Current gaps and challenges in anatomy education in Rwanda

The Ministry of Health in Rwanda initiated the 4 × 4 program to quadruple the number of healthcare workers in 4 years. To achieve this goal, the Ministry of Health and the Ministry of Education agreed with many institutions in Rwanda to increase the number of medical students in training. However, the increase in student numbers at many institutions was due to the absence of additional rooms and lectures on the staff. This led to a large number of students in one class, approximately 150 students in one class with one lecture. This also affects the anatomy lab, especially the dissection lab, where there is limited time as large numbers use the same laboratory^[2]^.

Although the anatomy staff includes specialists and surgeons, their involvement in teaching roles is still minimal. These professionals could share valuable clinical insights, practical experiences, and scenarios to help deepen students’ anatomy skills, but this potential remains untapped. Additionally, the complexity of anatomy modules requires more activities for better understanding, like workshops based on anatomy, which are still scarce in Rwanda^[1]^.

Team-based learning (TBL) is a program initiated by Prof. Dr. Julien Gashegu at the UR. It assesses students’ understanding of a given topic through group discussions and collaborative learning. However, it has been ineffective due to insufficient group discussions, unsupervised sessions, and limited time. This represents a significant gap in anatomical education, as students miss out on the benefits of collaborative learning and critical thinking^[11]^.

Cadaver dissection practice has become one of the challenges that anatomy education faces in Rwanda. As cadaver dissection is a gold standard for teaching anatomy, acquiring and maintaining cadavers is quite challenging. So far, there are no best strategies in place to acquire and preserve cadavers for teaching purposes, rendering anatomy education somehow tricky and not as effective as it was. Another challenge is a shortage of resources; as in many low- and middle-income countries, Rwanda faces resource constraints. Suitable equipment, facilities, and infrastructure are essential for effective anatomy education. Obtaining and maintaining educational resources such as cadavers, models, and digital tools is expensive^[12]^.

## Practices to improve anatomy learning

Anatomy is one of the most essential basic medical sciences and is critical in medical education, relevant to all healthcare professions^[13]^. Therefore, medical students need to acquire crucial anatomical knowledge to establish a strong foundation for their future clinical and qualified practices. Quality anatomy teaching and learning can significantly contribute to producing competent surgeons and healthcare professionals. An adequate number of qualified anatomy trainers is necessary to improve anatomy learning^[9]^.

Rwanda has a policy to quadruple the number of healthcare providers. However, increasing the number of healthcare providers without enhancing their anatomical skills could be counterproductive. Thus, qualified staff with an appropriate student-staff ratio is essential for improved anatomy learning^[2]^. In earlier years, specifically around 2003, anatomy was primarily taught through traditional face-to-face lectures, cadaveric dissections, and atlas pictures. These methods have been the cornerstone of anatomy education. However, researchers argue for a crucial shift from traditional to modern teaching methods for better educational outcomes^[14]^.

Modern methods of teaching anatomy include but are not limited to 3D atlases and mobile applications that allow learners to explore virtual bodies and identify spatial relationships interactively. Other contemporary techniques include plastination, which creates durable anatomical specimens, 3D printing, simulation, virtual reality, virtual dissection, social media, and radiology technologies. These should be incorporated into anatomy teaching to enhance learning outcomes^[15]^.

Moreover, increasing resources for anatomy education is another strategy to improve learning results. Adequate equipment and financial resources are essential. Laboratories should be well-equipped, and libraries should have a sufficient number of updated books to support the education of future healthcare providers^[2]^.

## Impact of improved anatomy education on health care delivery

Anatomy education plays a crucial role in preparing future healthcare providers. In biomedical sciences, students engaging with anatomy modules enhance their understanding of the human body, correlating the medical knowledge with anatomical functions of certain parts and understanding the different variations among the population and others. Collectively, this improves their ability to deliver effective health care. Solid anatomical knowledge is essential in the medical education sector, where understanding the structures and positions of internal body parts and their associated pathological implications, as well as their treatment modalities, is imperative for effective healthcare promotion^[16]^.

Furthermore, improved anatomy education significantly contributes to positive and long-lasting surgical outcomes, and surgeons must comprehensively understand anatomical structures and arrangements before performing any surgical procedures and operations. This knowledge from anatomy education improves the performance of the surgeons in the operating room, reducing risks of harm during operations, improving surgical patients’ satisfaction overall, and improving healthcare. At the UR, surgical residents across various departments, including ENT, general surgery, obstetrics and gynecology, urology, plastic surgery, and neurosurgery, have reported better outcomes, partly attributable to their reinforced understanding of anatomical principles which is a positive indicator for the role of improved anatomy on the surgical implications^[17]^.

Anatomy education also aids in the professional identity formation of students in the health care sector, positively influencing the clinical practice of health care providers; anatomy modules are among the biomedical modules that are taught to undergraduate students so its improvement would improve the effective medical education training; the contributions from anatomy education extend to various areas, enhancing competencies and improving patient care^[18]^.

Additionally, improving anatomical research education is paramount for identifying anatomical variations, which can serve as critical references in diagnosing regional diseases and understanding their underlying causes. Different comprehensive reports on anatomy research have shown significant contributions to enhancing healthcare systems worldwide through different healthcare advancements, including research. Improved anatomy research results in the development of policy implications such as variations correspondence, surgical operations development, and others^[19]^.

## Recommendations for the future

Quality anatomy delivery plays a crucial role in strengthening the health care system. For quality delivery, a functioning anatomy unit is needed. This unit requires a guiding philosophy, qualified staff with an adequate student-to-staff ratio, adequate infrastructure and equipment, adequate financial resources, and proper teaching and learning methods^[2]^. On July 13, 2023, the Cabinet of the Government of Rwanda approved the 4 × 4 reform as a national priority. This reform aims to quadruple the number of healthcare workers in the next 4 years while enabling the country’s health sector and medical education to evolve and be sustainable, There will also be a high demand for the workforce to equip many students with high-quality knowledge^[20]^.

To cope with this, there should be proper learning strategies based on student involvement complemented with other approaches like cadaver-based approaches and electronic labs, like peer learning programs where students come together and share knowledge academically and experiences^[15,21]^. There is also TBL, where students discuss the content in advance and take an assessment to evaluate their understanding points and determine where to improve during lectures. This collaborative approach towards quality anatomy delivery has been productive, and there is room for improvement to achieve a sustainable healthcare system in Rwanda^[11]^.

Currently, a peer learning program in different modules, including Human anatomy, has been implemented by the Medical Students Association Rwanda through its Standing Committee on Medical Education. The program has been productive, as medical students revealed through their feedback. It should be more practical with frequent utilization of practical resources than emphasizing a theoretical approach. This should be put in the anatomy modules calendar and supervised by qualified staff members to assist students with content delivery, evaluate the progress, and ensure quality content delivery^[2]^.

Surgeons and other relevant clinicians should also be involved, as they can easily extract clinical applications from their own experience, and students’ interaction with those in practice is very motivating. Better education should not only be about acquiring knowledge of facts but also about experiencing personal and professional transformation and practicing new skills. Learning gets better when students are actively engaged in the process^[22]^.

## Conclusion

Anatomy education equips essential knowledge and skills and is a pivot for strengthening Rwanda’s health care system. A better understanding of anatomy leverages accurate diagnosis, effective treatment planning, and successful surgical interventions, which improves the quality of patient care and thus leverages the health system. Some challenges are reported in improving anatomy education, including low engagement, inadequate department funding, and inadequate workshops and engagement in anatomy learning. Younger students are not engaged in anatomy research, which must also be promoted.

Different teaching models, such as TBL and peer learning programs, need to be promoted, and modern technologies need to be used mostly by medical students in anatomy education, enriching the experience for them. This will embark on a 4 × 4 reform to sustain Rwanda’s health care system. Promoting continuous learning and collaboration among students and educators would help prepare a competent and skilled healthcare workforce ready to tackle emerging health challenges. Promoting anatomy education is a critical step toward advancing Rwanda’s population’s overall health and well-being.

Additionally, investing in anatomy education is not only necessary for individual health professionals but also a critical step toward advancing the overall health and well-being of the Rwandan population.

## Data Availability

Not applicable.

## References

[1] Ndahimana P Ngenzi J Izabayo J. Students’ perception toward blended anatomy learning modalities in the COVID-19 era. Rwanda Med J 2023;80:102–11.

[2] Gashegu J. Developing an anatomy unit in Rwanda: overcoming challenges. Rwanda Med J 2023;80:112–17.

[3] (N.d.-b). Gov.Rw. Retrieved August 16, 2024, from https://www.moh.gov.rw/fileadmin/user_upload/Moh/Publications/Reports/Health_sector_performance_Report_FY_2019-2020.pdf

[4] Carmichael H Coleman JR Samuels JM. “Bedside Anatomy”: a tool to contextualize learning and introduce surgical careers. J Surg Res 2020;249:1–7. 31911140 10.1016/j.jss.2019.12.015

[5] Asante EA Maalman RS Ali MA. Perception and attitude of medical students towards cadaveric dissection in anatomical science education. Ethiop J Health Sci 2021;31:867–74. 34703187 10.4314/ejhs.v31i4.22 PMC8512940

[6] Asad MR Mutairi AA AlZahrani RE. Role of living anatomy in medical education: a narrative review. J Pharm Bioallied Sci 2023;15:S843. 37694003 10.4103/jpbs.jpbs_235_23 PMC10485445

[7] The significance of anatomy as a core subject in medical training and knowledge. 2023. GS Medical College & Hospital. https://gsmedicalcollege.in/the-significance-of-anatomy-as-a-core-subject-in-medical-training-and-knowledge.php

[8] Streith L Cadili L Wiseman SM. Evolving anatomy education strategies for surgical residents: a scoping review. Am J Surg 2022b;224:681–93. 35180995 10.1016/j.amjsurg.2022.02.005

[9] Almizani MS Alotaibi MA Bin Askar MF. Clinicians’ and students’ perceptions and attitudes regarding the anatomical knowledge of medical students. Adv Med Educ Pract 2022;13:1251–59. 36225717 10.2147/AMEP.S370447 PMC9549804

[10] Linghu E. A new stage of surgical treatment: super minimally invasive surgery. Chin Med J (Engl) 2022;135:1–3. 34074847 10.1097/CM9.0000000000001534 PMC8850819

[11] Sibomana I Karenzi ID Niyongombwa I. Use of student-generated multiple choice questions to enhance team-based learning of anatomy at the University of Rwanda. Adv Med Educ Pract 2020;11:825–32. 33177911 10.2147/AMEP.S274298 PMC7650038

[12] Naidoo N Akhras A Banerjee Y. Confronting the challenges of anatomy education in a competency-based medical curriculum during normal and unprecedented times (COVID-19 pandemic): pedagogical framework development and implementation. JMIR Med Educ 2020;6:e21701. 32873536 10.2196/21701 PMC7546732

[13] Bahadoran H Dadfar R Asadi MH. The role of anatomy in medical education. Asj 2022;19:51–58URL.

[14] Leung BC Williams M Horton C. Modernizing anatomy teaching: which resources do students rely on? J Med Educ Curric Dev 2020;7:238212052095515. 10.1177/2382120520955156 PMC7649918 33225067

[15] Darras KE Spouge R Hatala R. Integrated virtual and cadaveric dissection laboratories enhance first-year medical students’ anatomy experience: a pilot study. BMC Med Educ 2019;19:1–6. 31590672 10.1186/s12909-019-1806-5 PMC6781397

[16] Khan IA Singh Y. The crucial role of anatomy in shaping competent medical doctors. Indian Pract 2023;76:33.

[17] Gashegu J Hakizimana D Ncogoza I. Impacting surgical training: role of surgical anatomy dissection courses. Rwanda Med J 2023;80:91–101.

[18] Mat Nawi NF Hadie SNH. Interprofessional anatomy education: its significance, challenges, and recommendations. Educ Med J 2024;16:165–76.

[19] Mahara G Tian C Xu X. Revolutionizing health care: exploring the latest advances in medical sciences. J Glob Health 2023;13:03042. 37539846 10.7189/jogh.13.03042 PMC10401902

[20] *The 4x4 reform: A path to quality health care in Rwanda*. (n.d.). Gov.Rw. Retrieved August 16, 2024, from https://www.moh.gov.rw/news-detail/the-4x4-reform-a-path-to-quality-health-care-in-rwanda

[21] Zamiri M Esmaeili A. Methods and technologies for supporting knowledge sharing within learning communities: a systematic literature review. Adm Sci 2023;14:17.

[22] Hashemiparast M Negarandeh R Theofanidis D. Exploring the barriers of utilizing theoretical knowledge in clinical settings: a qualitative study. Int J Nurs Sci 2019;6:399. 31728392 10.1016/j.ijnss.2019.09.008 PMC6838863

